# Blood–brain barrier: emerging trends on transport models and new-age strategies for therapeutics intervention against neurological disorders

**DOI:** 10.1186/s13041-022-00937-4

**Published:** 2022-06-01

**Authors:** Hema Kumari Alajangi, Mandeep Kaur, Akanksha Sharma, Sumedh Rana, Shipali Thakur, Mary Chatterjee, Neha Singla, Pradeep Kumar Jaiswal, Gurpal Singh, Ravi Pratap Barnwal

**Affiliations:** 1 Department of Biophysics, Panjab University, Chandigarh, 160014 India; 2 University Institute of Pharmaceutical Sciences, Panjab University, Chandigarh, 160014 India; 3 Department of Biotechnology, UIET, Panjab University, Chandigarh, 160014 India; 4 Department of Biochemistry and Biophysics, Texas A&M University, College Station, TX 77843 USA

**Keywords:** Blood–brain barrier, BBB dysfunction, Drug delivery, Nanoparticles, Central nervous system, Neurological diseases

## Abstract

The integrity of the blood–brain barrier (BBB) is essential for normal central nervous system (CNS) functioning. Considering the significance of BBB in maintaining homeostasis and the neural environment, we aim to provide an overview of significant aspects of BBB. Worldwide, the treatment of neurological diseases caused by BBB disruption has been a major challenge. BBB also restricts entry of neuro-therapeutic drugs and hinders treatment modalities. Hence, currently nanotechnology-based approaches are being explored on large scale as alternatives to conventional methodologies. It is necessary to investigate the in-depth characteristic features of BBB to facilitate the discovery of novel drugs that can successfully cross the barrier and target the disease effectively. It is imperative to discover novel strategies to treat life-threatening CNS diseases in humans. Therefore, insights regarding building blocks of BBB, activation of immune response on breach of this barrier, and various autoimmune neurological disorders caused due to BBB dysfunction are discussed. Further, special emphasis is given on delineating BBB disruption leading to CNS disorders. Moreover, various mechanisms of transport pathways across BBB, several novel strategies, and alternative routes by which drugs can be properly delivered into CNS are also discussed.

## Introduction

The central nervous system (CNS), which includes the brain, is considered as the most important part of the entire human body and is often referred to as the controlling center of the body. Neurons are vital components of the CNS and these neural networks are responsible for regulating neuronic signaling by employing various electrical and chemical signals, which thus regulate the ionic environment between the axons and synapses [1]. A well-developed organism has a primary interface between the CNS and the outer region of the body referred to as the “blood–brain barrier” (BBB), which was discovered and named in the early twentieth century. This barrier plays a crucial role in regulating the optimal neural environment and maintaining homeostasis [2–4]. BBB between the two compartments of blood circulation and CNS comprises of various complex multicellular structures. The characteristic features of these structures selectively permit or restrict the transition of substances. There exist two distinguishable barriers between blood and CNS, referred to as the thin endothelial BBB and the epithelial “blood to cerebrospinal fluid (CSF) barrier (BCSFB)”. The endothelial BBB is localized in all the layers of cerebrospinal tree, whereas the epithelial blood-CSF is positioned in the brain ventricular system [5]. The distinctiveness of the microvasculature of the CNS in the presence of non-fenestrated vessels as well as other additional components help in strictly controlling the influx and efflux of various molecules, ions, and other important cells across the blood and brain barrier [6].

The BBB is present as a highly selective semi-permeable interface between blood and brain, possessing a defensive network of blood vessels and brain tissues that are complex, dynamic, and prevent the entry of harmful substances such as neurotoxic debris derived from blood, cells, and other microbial pathogens [6]. Effectively blocking the penetration of these substances from blood into the CNS by this route is essential to prevent the initiation of neurodegenerative conditions. This barrier consists of various neurotransmitters that help in effectively communicating with other cells of the CNS for regulating crucial events and maintenance of homeostasis. For instance, they act in response to pathological conditions; during the beginning as well as the progression of disease [4, 6]. Stem cell therapy is emerging as a promising treatment modality against various CNS disorders. Neural stem cells ensure the proper functioning of the brain and BBB along with maintaining homeostasis. These stem cells synthesize gene products with therapeutic properties ideal for treating neurodegenerative disorders [7]. This therapy is being used for ischemic stroke [8], AD [9], and which are even for brain cancer (using stem cells derived from bone marrow) [10]. It is trusted that in conjunction with other therapies for various CNS diseases, it will prove to be a breakthrough.

The BBB acts as an asset by securing the brain, however, on the other hand, there are certain challenges associated with it. The discovery and design of therapeutic agents or treatment for life-threatening brain disorders has been a major challenge for decades. The purpose of referring to BBB as a challenge highlights that along with preventing entry of harmful or toxic substances across this barrier, it hinders the uptake of neuro-therapeutic drugs important for treating patients suffering from CNS disorders [11]. These brain-linked diseases include cerebral ischemia, brain trauma, multiple sclerosis (MS), Alzheimer’s disease (AD), tumors [12], Huntington’s disease, amyotrophic lateral sclerosis (ALS), Parkinson’s disease (PD), hippocampal sclerosis, α­synucleinopathy, and prion disease etc. [4, 13–16]. The significance of BBB is well understood and research over the past years has delineated various essential functions of this barrier in the regulation of ions using specific ion channels and transporters for reliable synaptic signaling by maintaining the neutral ion composition [1]. In CNS, the barrier ensures non-synaptic signaling by separating central and peripheral neurotransmitter pools via reducing crosstalk among them. The BBB and the BCSFB, together effectively direct the movements of ions such as Ca^2+^ and Mg^2+^, and maintain pH in the CNS. BBB regulates homeostasis, which is indispensable for the proper functioning of neurons and neural signaling [17, 18]. It guards the brain by keeping out toxins, pathogens and helps in maintaining low protein concentration in the CNS environment [6]. Further, the uptake of neurotoxic substances in the blood, for instance, xenobiotics, proteins, metabolites, etc., through the environment or food is forestalled by BBB. To ensure the safety of the brain, BBB serves as a shield against the entrance of such aforementioned neurotoxins into the brain [1]. This barrier also ensures minimum inflammation by regulating the entry of leukocytes through this route and prevents brain injuries [19]. Further, serious pathological consequences due to the passage of large macromolecules like giant serum proteins into the brain can cause damage to BBB. For example, plasma proteins like albumin and plasminogen damage the nerve tissue and in interstitial fluid (ISF), thrombin and plasmin initiate a cascade leading to seizures, scarring, glial cell division, and cell death, etc. Therefore, BBB confines the passage of such macromolecules cautiously [1].

Moreover, research over more than 130 years has shown that BBB apart from being a barrier can also act as a carrier. The BBB is referred to as a carrier because of its ability to transport the necessary nutritional molecules like vitamins, minerals, glucose, lipid-soluble molecules, and gases such as carbon dioxide and oxygen present in the blood to the brain as well as helping the elimination of toxins/metabolites [3]. Interestingly, it is worth noticing that BBB as a carrier is extremely pivotal since glucose (also called fuel for the brain) transportation is vital for the appropriate functioning of the human brain and around 20% of the total body energy is used by the brain to work effectively [3, 6].

This review provides insights into various significant aspects of BBB. The details regarding major structural components are well explained followed by an outline of activation of the immune response as this barrier is breached. Further, various autoimmune and other neurological disorders caused due to BBB dysfunction have been discussed. Insights regarding proposed mechanisms for permeation and transport pathways across BBB for effectively targeting the brain by bioactive substances through several novel strategies including invasive systems, non-invasive/miscellaneous systems, and alternative routes for drug delivery to CNS are also provided. In addition, different factors that hinder the uptake of neuro-therapeutics to treat life-threatening-brain diseases of CNS are described. Moreover, information about different nanoparticles that are being used for the effective delivery of drugs to the CNS and the potential of nanoparticle-based approaches for treating neurological disorders has also been discussed.

## BBB: structural details

**Fig. 1:**
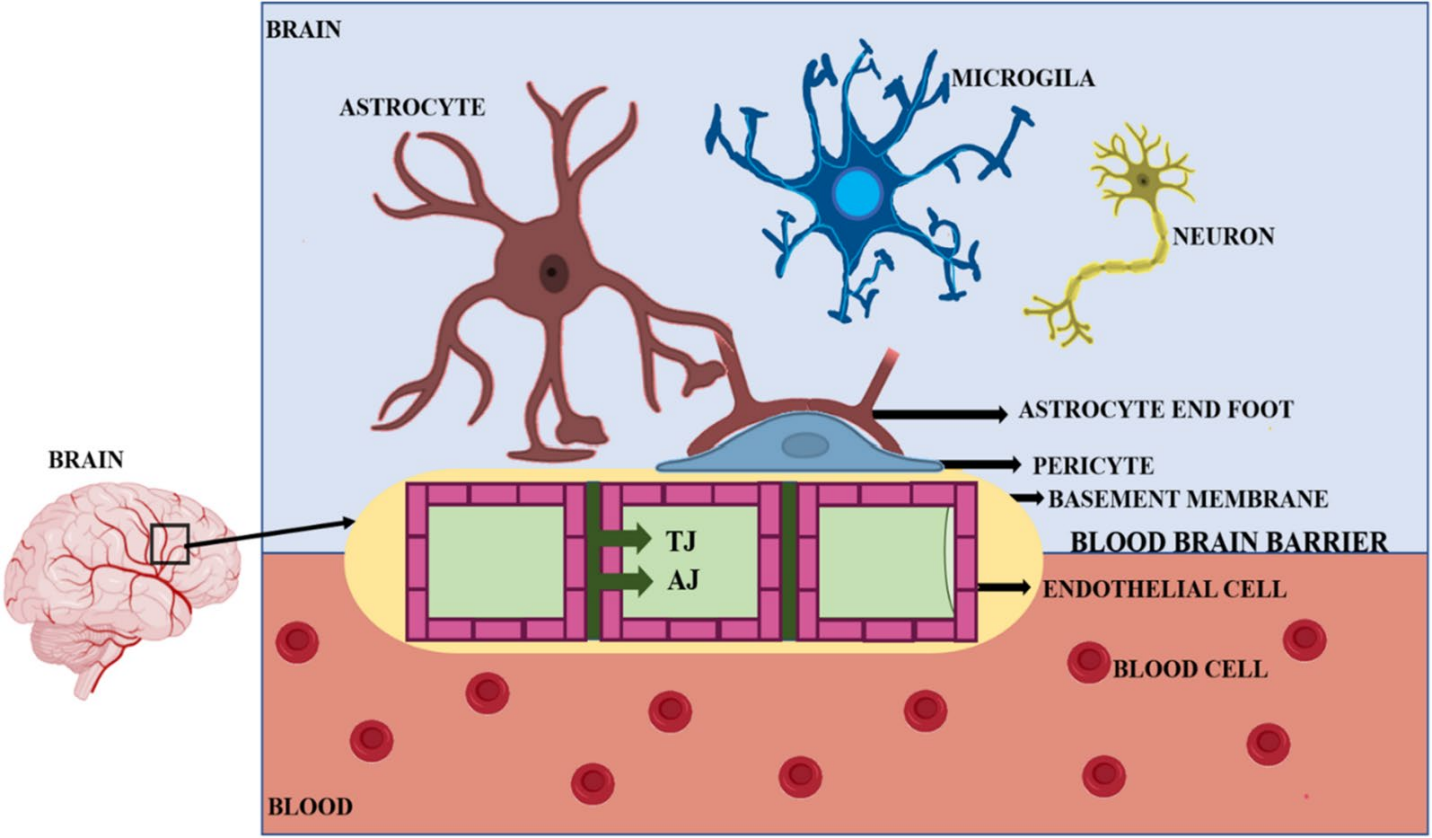
Blood–brain barrier (BBB) building blocks: endothelial cell (ECs), basement membrane, pericytes, astrocytes, adherent junction (AT) and tight junctions (TJ)

The BBB primarily functions as a defense line that helps to control the internal brain environment. The principal components that form this barrier are cerebral endothelial cells (ECs), pericytes, astrocytes, and basement membrane as shown in Fig. 1 [1, 4, 19, 20]. The blood vessel walls are formed by the ECs. The ECs of the capillary walls are tightly connected without any gap and lack pinocytic vacuoles; further, tight junctions (TJs) and adherent junctions (AJs) present in between these ECs permit them to tightly control the passage of undesirable substances and pathogens between blood and brain. The ECs of the CNS are highly selective and only allow the molecules with appropriate mass and lipophilicity to pass through [20]; while the ECs involved in circulation possess gaps and subsequently, the exchange of substances is comparatively easy. ECs tightly held by TJs (zonulae occludentes) and AJs act as a physical barrier at the interface which furthermore provides structural support to BBB [20].

The key feature of these TJs is to restrict the permeation of macromolecules, ions, and other polar solutes through paracellular diffusional pathways [1, 21]. TJs are comprised of various proteins traversing the intercellular cleft such as occludins and claudins, which are further linked to important scaffolding proteins as well as other regulatory proteins like cingulin, ZO-1, ZO-2, and ZO-3 [21]. In vivo studies reported that out of more than 20 known isoforms of claudins, the absence of claudin-5 and claudin-3 lead to loss of BBB integrity and barrier disruption, which, in turn, disturbs the proper functioning of BBB [1, 21, 22]. Apart from forming and maintaining this barrier, TJs also play key role in organization and regulation of interaction among these proteins [1, 19], while AJs provide underlying support to tissues by holding the cells together at the junctional complexes present in between ECs. AJs consist of proteins such as cadherins, the junctional adhesion molecules (JAMs)-JAMC6 and JAMB, platelet endothelial cell adhesion molecule (PECAM-1) etc,. which are also significant for TJs formation along with BBB maintenance [1, 13, 22].

Additionally, the ECs of CNS experience a low rate of transcytosis resulting in lowering the rate of the exchange facilitated by vesicle mediated transcellular transport as compared to peripheral ECs [1, 21]. Further, to regulate the CNS homeostasis, the CNS ECs utilize two main categories of transporters named efflux transporters and specific nutrient transporters [6, 23]. The efflux transporters transport a wide range of lipophilic molecules, whereas nutrient transporters ensure the supply of specific nutrients across the BBB and help in the removal of waste products by their transport from CNS into the blood [6, 24]. Some essential efflux transporters are Mdr-1 (P-glycoprotein), breast cancer resistance protein (BCRP), and multidrug resistance-associated proteins (MRPs) [6]. Whilst, those belonging to CNS ECs for the delivery of nutrients into the CNS parenchyma include slc2a1GLUT1 required for transportation of glucose, slc7a1 for transport of cationic amino acids, slc16a1 and l-DOPA for supplying lactate and pyruvate and slc7a5 for the purpose of transporting neutral amino acids [6, 16]. Besides, Leukocyte adhesion molecules (LAMs) are also expressed by CNS ECs to control immune cell entry in the CNS [1, 6, 25].

In the BBB structure, the ECs are covered by pericytes from the outside of the blood vessel. Pericytes are derived from neural crest as well as mesoderm and are lined on the ablumenal surface of the micro vascular endothelial tube [26]. These cells consist of contractile proteins possessing the ability to contract the capillary diameter as needed [6, 27]. The paramount significance of these cells includes their roles in maintaining blood flow in response to neural activities, for directing the formation of blood vessels, i.e., angiogenesis; postnatal formation of BBB, and healing of wounds etc. Moreover, pericytes are also responsible for controlling the proper formation of BBB during the time of development in adulthood, during aging and in the regulation of its effective functioning [6].

Astrocytes are star-shaped cells, also referred to as astrocytic glial cells located on the basal lamina at the parenchymal side; having end-feet projections through which they interact with the ECs [28]. In astrocytes*,* the astrocytic end-feet projections are critical to provide biochemical support required for the maintenance of TJs as well as the ECs [29]. They also intimately promote the formation of TJs present in between the ECs. Additionally, astrocytes also consist of various proteins like dystroglycan, dystrophin, and aquaporin 4 (major cerebral water channel); the former two proteins essentially take part in linking end feet anatomical structure to the basement membrane [30, 31]. Astrocytes ensure the appropriate supply of various essential nutrients  for nerve tissues [25, 29]. For instance, the astrocytes near the neurons behave as a glucose storage unit and regulate sufficient release of glucose to neurons in case of shortage. Apart from this, the pivotal role of these cells is not simply associated with repairing and scarring process in case of brain injuries caused due to trauma, but also to safeguard the CNS by removal of waste or harmful metabolic substances [29, 32, 33].

The basement membrane is another important component which is made up of astrocytes, pericytes as well as ECs enclosing the CNS [34]. Two types of basement membranes are present at the BBB, one secreted by pericytes and ECs, namely the vascular basement membrane, also called endothelial basement membrane, and the other formed by astrocytes, referred to as the glial basement membrane (also known as parenchymal basement membrane) [35, 36]. The vascular and glial basement membranes have different compositions and these membranes join and separate to accommodate different fluid secretions and cells [37]. Both basement membranes surround the vascular tube and have different molecules. The vascular basement membrane is comprised of four different glycoprotein families including laminins, nidogens, heparan sulphate proteoglycans, and collagen type IV [36]. Matrix metalloproteinases (MMPs) interrupt the proper working of these glycoproteins, leading to the dysfunction of BBB [6].

Different cell adhesion molecules (CAMs) promote the migration of leukocytes to the CNS resulting in traversing across the ECs, leukocytes cross the basement membranes to gain access to the CNS [6]. Different cells like ECs, pericytes, and astrocytes secrete several structural proteins like fibronectin, collagens, laminins etc., for which the basement membrane serves as the extracellular matrix [38, 39]. An essential role is played by the laminin in maintaining the integrity of CNS components, especially the endothelial basement membrane. This further has an impact on the migration of T lymphocytes. The interaction of laminins with T lymphocytes can thus be targeted against various inflammatory processes in the CNS [36, 40].

## BBB and functioning of the immune system

Until recently, it was postulated that the lymphatic vessels are altogether absent in the brain and BBB along with BCSFB has been together known to constitute the CNS into an immune-privileged organ, which means that immune cell entry does not take place in the CNS; however, several studies have reported that the T cells gain access to the CNS during disease conditions [41, 42]. Recently, the glymphatic model has been discovered in many organisms including humans. It is being extensively studied by comparison between normal and disease conditions (neurological disorders like ischemic stroke, AD, etc.). Changes in the glymphatic system and the BBB are associated with the occurrence of many neurodegenerative diseases [43]. The glial lymphatic system (termed as glymphatic system) is linked with a classic network of lymphatic vessels and connected with the meninges layer covering the brain as well as nerves and blood vessels [44, 45]. Ageing has been associated with reduced diameter of meningeal lymphatic vessels which plays significant role in accumulation of various proteins involved in neurodegeneration [45].

Tau and β-amyloid are known to exit the brain by means of the glymphatic system. This system also eliminates other harmful metabolites from the CNS, crucial for efficient functioning of the CNS [46]. Patients suffering from AD experience reduced functioning of the glymphatic system as well as the BBB. This leads to the neurovascular unit (NVU) dysfunction, including pericyte degeneration and reduces clearance of tau and β-amyloid [47].

**Fig. 2:**
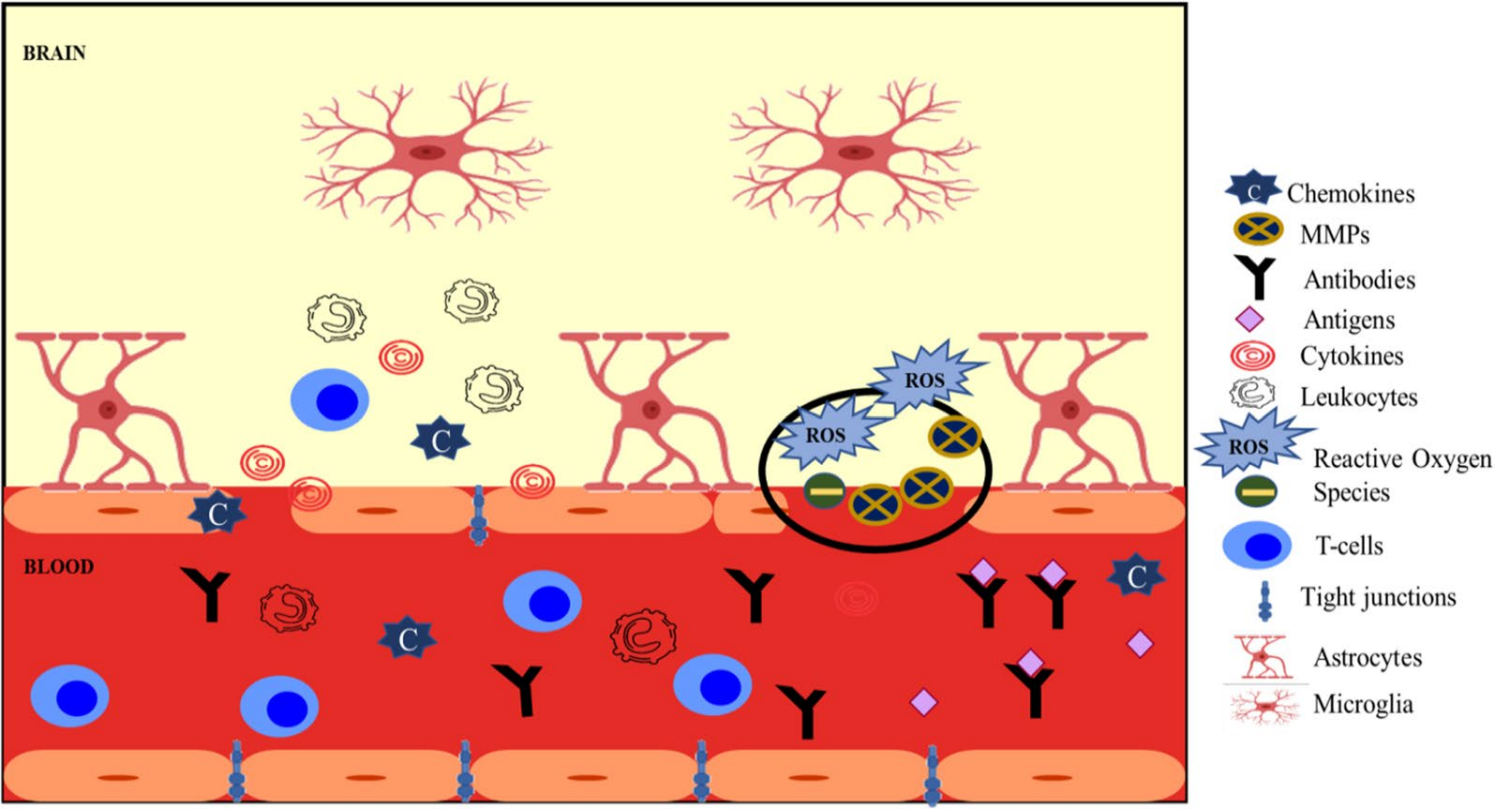
Breaching of the BBB and entry of effectors of the immune system. Rupturing of the blood vessel is followed by leukocyte release; cytokines like IL-1, IFN-γ, TNF-α and chemokines are also secreted by endothelial cells (ECs) joined together via tight junctions (TJs). Vessel rupture promotes release of reactive oxygen species (ROS) and matrix metalloproteinases (MMPs), resulting in basement membrane disintegration. The figure has been adapted from [57]

In light of intrusion by a pathogen, the immune response generated in the host involves the production of a wide repertoire of cells, all of which have significantly different roles. In the event of a pathogen invasion; firstly, the innate immune system reacts, followed by mediators of adaptive immunity. The innate immune response concerns every cell present in the CNS and is more prevalent than adaptive immunity. Macrophages, microglia, and mast cells regularly interact with the BBB and primarily act against pathogen invasion [6, 48]. Microglia reside in the CNS and play a major role in the innate immune response and secrete various cytokines like interferons (IFN γ), Interleukin 1 (IL-1), Tumor Necrosis Factor (TNF)-α and chemokines besides MHC Class II molecules. These also control neural development. The enhanced production of microglia during diseases like AD and MS is responsible for BBB damage [6, 49, 50]. These cells contact the encompassing neurons and promote the phagocytosis of pathogenic cells, subsequently restoring the homeostatic balance [51]. Mast cells, derivatives of hematopoietic stem cells are also constituents of the innate immunity and produce various inflammatory molecules. Their differentiation begins in bone marrow, but they circulate in the blood before migrating to various tissues where differentiation is completed [52]. They contribute significantly to tissue repairing and also mount allergic reactions. These cells have been reported for their potential role in various neurological disorders [53, 54]. It has been reported that some chemokines including CCL_19_, CCL_20_, CCL_21,_ and a few other control the leukocyte entry inside the CNS [55, 56]. A pictorial depiction of the immune response associated with disruption of BBB [57] is given in Fig. 2.

Even though it was believed that most of the immune cells and other soluble molecules cannot pass between the CNS and other organs because of BBB and it protects the CNS from inflammation caused due to pathogen’s access to the host, it is known that the CNS has to be regularly checked for its proper functioning to ensure the absence of any injury or damage because of some pathogen [34, 58]. Macrophages and mast cells present in the CNS perform surveillance functions along with memory T cells that are present in the CSF [37]. The CSF drains many parts of the CNS and in a healthy person has lesser immune cells but during inflammation, B and T lymphocytes as well as antigen presenting cells (APCs) are abundantly present in CSF [59].

It is reported that BBB functioning is affected even before the immune cells migrate to the site of inflammation [60]. On interacting with the pathogen, microglia gather at the site of infection and generate reactive oxygen species (ROS), various cytokines, and chemokines like TNF-α, Monocyte chemotactic protein, IL-6, etc. [52, 61]. These attract leukocytes to the site, which activate the adaptive immune response. The ECs release various CAMs and pro-inflammatory cytokines. The interaction of leukocytes with ECs is made possible by molecules like CAMs and integrins etc. This leads to leukocyte movement across the BBB, often called Trans Endothelial Leukocyte migration (TEM) [62] which might be due to less stringent adhesion of the TJs among ECs. This interaction may aid the microglia cells already present in the CNS in managing disease conditions [63]. In addition, the production of different inflammatory molecules can also stimulate astrocyte activity. This cascade of events can damage the neurons and bring about irreversible changes in the BBB. Stimulation of the immune system for a long time, particularly glial cells can result in sustained inflammation and neurodegenerative changes in the CNS, paving way for the onset of diseases like MS, AD, PD, etc. [52, 64, 65].

## BBB dysfunction

BBB disruption and dysfunction in a pathological state prompts the leakage of blood constituents into the CNS, infiltration of various cells, abnormal transfer and clearance of ions and molecules responsible for reduction and dysregulation of cerebral blood flow (CBF), further causing various neurological deficits [66].

### Acute neurological diseases due to BBB dysfunction

#### Bacterial meningitis

Bacterial meningitis refers to inflammation of the subarachnoid space and meninges caused by various bacterial pathogens viz., *Streptococcus pneumoniae, Neisseria meningitidis, Haemophilus influenzae*, and *Escherichia coli* [67]. The inflammation also involves the brain cortex and spinal cord [68]. Bacterial invasion in the brain takes place through four processes, which include colonization, bacterial intrusion into the blood flow, survival in blood, and penetration into the subarachnoid space [28]. The consequent inflammation and CNS damage are brought about by a blend of pathogen and host factors. The primary manifestations associated with bacterial meningitis include pyrexia, cephalalgia, migraine, photophobia, and change in consciousness [69].

Blood-borne bacteria can subjugate the meninges through the choroid plexus (CPs) and CNS barrier capillaries which include the arachnoidal, pial, and brain parenchyma microvessels. *H. influenzae* interacts with the basolateral side of CPs ependymal monolayer and transcytoses to the cytoplasm and is released in ventricles by exocytosis. *H. influenzae* proficiently adheres to ECs [70]. Group B Streptococcus (GBS) and *E. coli K1* are the two main causative agents of neonatal meningitis but both have different strategies of invasion in the subarachnoid space. Some *E. coli K1* factors like AB-type toxin, cytotoxic necrotising factor-1 (CNF1), invasion of brain endothelial cells (IBE) aid bacterial invasion of ECs that actuate RHO family GTPases [71]. Interaction of bacterial factors with host cell receptors triggers various host cell signaling proteins [28]. Factors like Outer-Membrane Protein A (OmpA) mediate adhesion through N-acetyl glucosamine of the glycoprotein (GP96), FimH and the lipoprotein NLPI permit adhesion of bacteria to host cells [72]. *N.* *meningitidis* and *S. pneumoniae* have their natural niche in the human nasopharynx, from where they arrive in the respiratory tract and enter the bloodstream and cause septicemia [71]. Virulence factors of *N. meningitidis* allow its survival in blood and prevent its elimination by host effector cells. Type IV pili (T4P) are essential for adhesion to  ECs. *N. meningitidis* adheres to CNS capillaries and actively crosses BBB by disrupting cell–cell junction in subarachnoid space [28].

*Streptococcus pneumoniae* can invade into the bloodstream through cavities of inner ear, intravascular space present within the tissues, or lungs. Once *S. pneumoniae* becomes bloodborne, it can invade the meninges through the olfactory neuron. The virulence factors that enable *S. pneumoniae* to survive in blood and brain parenchyma include capsule, pneumococcal surface proteins (PsP), and pneumolysin, also common for some other pathogens causing meningitis [73]. Encapsulated *S. pneumoniae* is more impervious to phagocytosis and facilitates successful colonization. PsP permit the adhesion of bacteria to ECs with the help of pneumococcal pilus-1. Further, Platelet Endothelial Cell Adhesion Molecule (PECAM-1) and poly Immunoglobulin receptor (plgR) promote translocation through BBB [74].

Numerous meningeal pathogens affect BBB integrity by interfering specifically with AJs or TJs. GBS and *S. pneumoniae* directly secrete a pore-forming toxin, thereby altering BBB integrity. *E. coli K1*, *S. pneumoniae*, and GBS enhance the production of nitric oxide from ECs via inducible nitric oxide synthase (iNOS), thus disrupting BBB integrity [75]. Moreover, pathogen-derived toxins enhance inflammatory chemokines or cytokines expression in response to infection by the host and can adversely affect BBB function [76].

#### Epilepsy

The characteristic feature of epilepsy is the fallible and uncontrolled activity of either a part or of the entire CNS. A patient susceptible to epilepsy experiences attacks when excitability of the CNS transcends a certain critical threshold [73]. The main difference between seizure and epilepsy is that the former is a single occurrence while the latter is characterized by two or more provoked seizures [77]. Epilepsy has been categorized as Grand Mal, Petit Mal, and Focal Mal epilepsy. Grand Mal epilepsy is manifested as neuronal discharges in the entire brain. The person becomes unconscious and this state lasts for 3–4 min. Increased voltage and recurrence of electrical signals can occur over the entire cortex [78].

Petit Mal epilepsy is characterized by unconsciousness for 3–30 s and muscle contractions around the head. The brain wave pattern of Petit Mal epilepsy can be demonstrated by spike and dome pattern [79]. In vivo studies suggested it to be resulting from oscillations of inhibition and excitatory thalamic and corticothalamic neurons, which in turn, initiate Grand Mal epilepsy [80].

Focal Mal epilepsy arises from some localized organic lesion or functional abnormality which may be confined to a single area, such as brain scar tissue that pulls the adjacent neuronal tissue; a tumor that squeezes the region of the brain, or congenitally deranged local circuitry [78]. Epilepsy can be caused by strong emotional stimuli, traumatic lesions and alkalosis caused by over-breathing. Other symptoms observed in patients include increased microvascular density, disturbed GABAergic mechanisms [81], loss of TJs, IgG leakage in hippocampal resections [82], a short period of amnesia [83], sudden anxiety, discomfort, attack of abnormal rage, jerks, shock movements and difficulty in breathing [84].

BBB dysfunction emphatically relates with seizure frequency and is not related to neuronal loss. Epileptogenic injuries or seizures initiate the synthesis and secretion of proinflammatory molecules such as TNF-α, IL-1β, and High Mobility Group Box 1 (HMGB1) in glial cells which result in decreasing seizure threshold, which further contributes to seizure precipitation, and recurrence because of rapid changes in glutamate and γ-aminobutyric acid (GABA) receptor phosphorylation. This also leads to channelopathies which change intrinsic neural excitability [85].

**Fig. 3:**
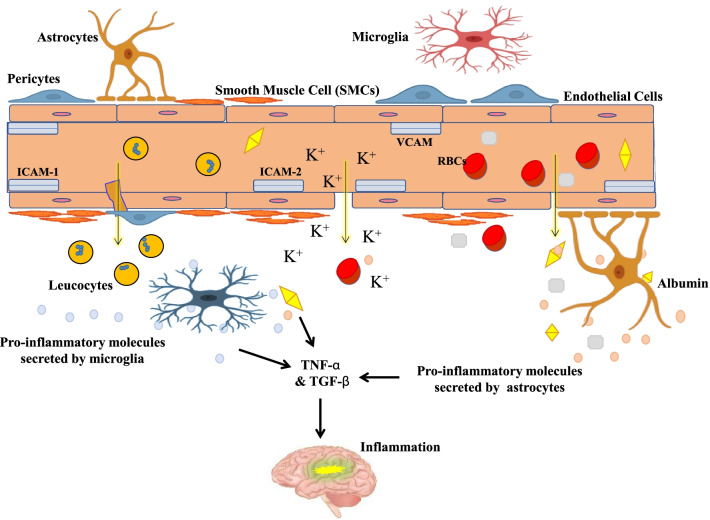
Activation of TNF-α and TGF-β Signaling by albumin during BBB breakdown induces rapid upregulation of genes associated to inflammation considering NF-ĸβ pathways and complimentary cascades, cytokines and chemokines (IL-6, CcL-2, CcD-7, Cd14) [86]. The pro-inflammatory molecules secreted by microglia and astrocytes contribute to voltage fluctuation in different parts of the brain. The figure is adapted from [87]

Seizures may also lead to BBB disruption, and artificial opening of BBB causes synchronization of rat neuronal activity that leads to albumin and immunoglobulin G (IgG) neuropil eruption. Albumin alters the buffering capability of K^+^ in astrocytes which contributes to neuronal hyperexcitability as shown in Fig. 3 [88]. TGF-β released from other cell types also affects BBB integrity. Astrocytic TGFβ released enhances plasminogen activator inhibitor-1 (PAI-1), which in turn suppresses tissue plasminogen activator (tPA), resulting in BBB closure [89].

#### Traumatic brain injury (TBI)

Traumatic brain injury (TBI), also known as acquired injury on the head/brain due to swift trauma harms the brain and disturbs the normal functioning of the brain, further causing substantial disability and mortality. It usually results from a powerful blow of an object or jolt (external force) to the head. CSF provides buoyancy and protects the brain from external forces, allowing the brain to bounce away from the skull when hit in the head, thus behaving like a shock absorber, but when this shock exceeds the threshold, as in the case of TBI, the levels of inflammatory proteins, like IL-6, IL-8 and IL-10 increase in the CSF [90]. The symptoms include loss of consciousness, post-injury amnesia, headaches, migraine, dizziness, light and sound sensitivity, seizures, blurred vision, anxiety, alterations in aggression, memory lapses, lack of impulse control, impaired decision making, and post-traumatic stress, etc.

BBB restricts the therapeutic compounds from entering the brain. However, in case of brain injury, ruptured microvessels depolarize the barrier which allows blood components to enter immediately into the brain parenchyma [91]. Primary injury caused due to dysfunction like neuroinflammation and cell death further disrupts the walls of these microvessels, initiating coagulation cascade and resulting in secondary damage [92]. Intravascular coagulation limits the supply of blood to tissues, which causes oxygen shortage, leading to ischemia, and thus, blood-borne factors fibrinogen, thrombin, and albumin among others can gain access to the brain [66]. Ischemia damages the blood components and may start inflammatory cascades which hinder the proper functioning of ECs, ECM, pericytes, and astroglia cells. BBB dysfunction due to TBI can occur in two phases: in the initial phase, shear injury of microvessels may occur within hours of TBI. The second phase is the activation of inflammatory cells after 3 days of TBI which trigger alterations in the BBB permeability. In vivo analysis reported the role of BBB breakdown in initiating changes to transcriptional processes of the neurovascular network, further causing degenerative disorders like AD, psychological impairments, cognitive decline, and epilepsy [92].

### Chronic neurological diseases

#### Alzheimer’s disease (AD)

**Fig. 4:**
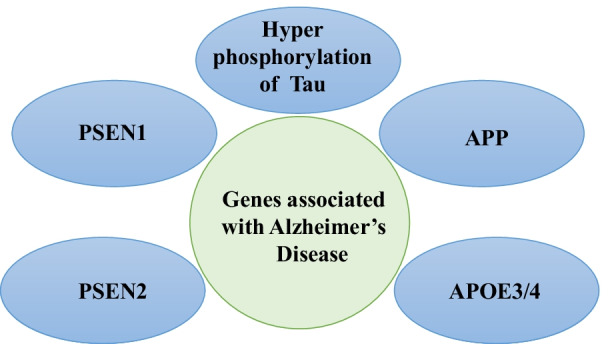
Schematic diagram showing mutations in several genes i.e. presenilin-1(PSEN1), presenilin-2 (PSEN2), amyloid-beta precursor protein (APP), apolipoprotein (APOE3), and apolipoprotein 4 (APOE4) related to increase risk of neurological disorders. Alzheimer’s disease (AD) is caused by mutations in the above genes and also by hyperphosphorylation of tau proteins in the distal part of axons in neuronal cells results in NFTs and cell death

AD is a brain disorder that leads to the progressive degeneration and death of brain cells. It results in a continuous decline in behavioral, thinking, and other social skills disrupting the person’s ability to function independently. Few symptoms of AD are dementia, anxiety, cognitive impairment, restlessness, fatigue, and dizziness, etc. [66]. Several genes like apolipoprotein (APOE4), presenilin-2 (PSEN2), presenilin-1(PSEN1), amyloid-beta precursor protein (APP), microtubule-associated protein tau (MAPT), etc., are also associated with a higher or lower risk of sporadic early or late-onset of AD (Fig. 4) [93]. The hyperphosphorylated tau neurofibrillary tangles (NFTs), Aβ plaques, neuronal loss, and cerebrovascular dysfunction are majorly proposed to contribute to AD pathophysiology and cognitive impairment [94]. NFTs caused by the accumulation of phosphorylated tau protein in the neuron can also lead to AD which is associated with chromosome no. 17q21. Moreover, tau is a microtubule-associated protein that facilitates axonal transport essential for neuronal signaling and trafficking [95]. In the normal brain, each tau molecule contains 2 to 3 phosphates, notwithstanding, the phosphoryl content increases by several folds in tauopathy patients. During hyperphosphorylation, tau protein dissociates from the microtubule, resulting in the spread of unbound microtubules and progressive accumulation of phosphorylated tau protein followed by the formation of NFTs [96]. On the other hand, PSEN1 and PSEN2 are the catalytic components of γ-secretase [97]. Mutations in human PSEN1 have been shown to promote the breakdown of BBB and cerebrovascular dysfunction [98]. PSEN2 mutations represent ~ 5% of all AD cases [99].

Besides, APOE4 is one of the major genetic risk factors leading to sporadic and late-onset AD. APOE4 alleles increase AD risk by four times, compared with APOE3. The cerebrovascular system and neurons suffer toxic effects due to APOE [100]. Human APOE4 carriers may develop progressive BBB breakdown and pericyte degeneration, [101] early neurovascular dysfunction, [102] and reduced glucose uptake by the BBB.

The vascular capillary leakages of proteins like thrombin, IgG, fibrinogen, albumin, and hemosiderin in the hippocampus and prefrontal as well as entorhinal cortex of the AD patients have been observed. Approximately 40 APP mutations and co-localization of proteins with Aβ [103] responsible for causing AD have been identified. This causes cerebrovascular pathology, breakdown of BBB and cerebral amyloid angiopathy (CAA) [104]. CAA is caused by vascular degeneration of smooth muscle cells (SMCs) linked with the breakdown of the BBB at the arterial and arteriolar levels [105]. Besides, APOE4 carriers accelerate BBB breakdown via activation of pathway i.e., proinflammatory cyclophilin A (CypA)-MMP-9. Thus, causing degradation of endothelial TJs, proteins of basement membrane and increasing damage to BBB [66].

#### Amyotrophic lateral disease (ALS)

Amyotrophic lateral sclerosis (ALS) causes dysfunction of neurons controlling voluntary movements. It is also referred to as motor neuron disease or Lou Gehrig’s disease. The patients suffering from ALS experience loss of hands and arm functions, difficulty in walking, speaking and even breathing. Respiratory failure can often be the reason for death and the average survival time after ALS diagnosis is around three years [106]. Upper Motor Neuron (UMN) and Lower Motor Neuron (LMN) are specific neurons affected by ALS. UMN extends from the cerebral cortex or brain stem and carries motor information, while LMN extends from the brain stem to the skeletal muscles to cause movement [107].

ALS can either be familial or sporadic. The frequency of sporadic ALS is random and accounts for around 90% of cases, while familial ALS is inherited. Around 10% of cases are familial with dominant inheritance of mutation in around 15 genes such as *TARDBP* (transactive response DNA binding protein), *ANG* (angiogenin), *FUS* (RNA binding protein), and *OPTN* (optineurin), etc. [108]. The respiratory symptoms of the disease include dyspnoea (breathing difficulties), orthopnoea (breathing difficulties while lying flat), weak cough, excessive sleepiness, and cognitive impairment, etc.

**Fig. 5:**
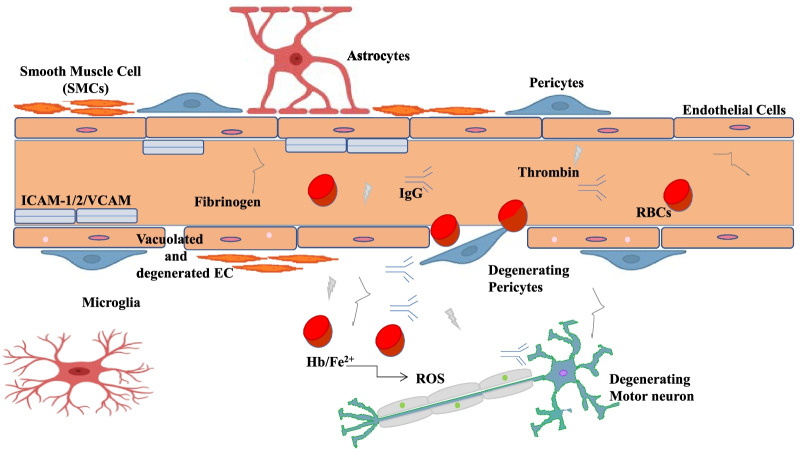
Reduced levels of TJs due to BBB dysfunction lead to pericyte degeneration causing infiltration of antibodies (IgG), intercellular adhesion molecule (ICAM-1/2/vascular cell adhesion molecules (VCAM), thrombin, plasminogen, haemoglobin (Hb) and iron (II) released from RBCs further produce ROS in the matrix, toxic for motor neurons in case of ALS. This figure is adapted from [66]

The BBB and BSCB restrict the entry of erythrocytes and plasma components into the CNS. The disruption of these barriers has been observed in ALS patients with spinal cord or motor-cortex accumulation of proteins like fibrin and thrombin [109], reduced levels of TJ proteins, erythrocytes, haemoglobin and hemosiderin, which further cause generation of ROS, toxic to motor neurons (Fig. 5). Levels of adhesion molecules such as intercellular adhesion molecule (ICAM-1/2/ vascular cell adhesion molecules (VCAM)) are increased in ALS. The elevated ratio of CSF/serum albumin and reduced expression of BSCB TJs proteins has been reported in both forms of this disease [110]. The key role of pericytes in maintaining blood-CNS barriers has been well understood.

#### Huntington’s disease (HD)

Huntington’s disease (HD) is a progressive disorder characterized by flicking movements in the muscles followed by severe distortional movements in the whole body. In addition, severe cognitive problems also develop along with motor dysfunctions. The genetics of HD abnormality involves a repeat sequence of CAG (≥ 36) in exon 1 of chromosome 4 of the *HTT* gene, producing mutant Huntington protein (mHtt) that aggregates and further causes neurodegeneration [66]**.** CAG encodes for the amino acid glutamine in the molecular structure of an abnormal neuronal cell protein Huntington responsible for the symptoms; thus, it can be considered a polyglutamine disease. A normal individual has 10–35 repeats of CAG and a higher number of CAG repeats leads to the onset of symptoms in the individual [111].

HD causes gradual degeneration of the basal ganglia, also called the caudate nucleus and putamen [112]. The symptoms associated with HD are chorea (jerky involuntary movements that affect the hips, shoulders, and face), cognitive decline, dystonia (abnormal muscle tone which results in muscular spasm and posture abnormality), and behavioral difficulties [113]. A reduction in expression of proteins like claudin-5 and Occludin involved in the formation of BBB TJs and various markers such as IL-8 and metalloproteinase 1 tissue inhibitor are related to increased BBB permeability, which have been observed in HD patients [114, 115]. BBB breakdown in HD patients has been confirmed by detecting 2.5-fold increase in levels of extravascular fibrin deposition [103] as compared to controls. Recent studies of dynamic contrast-enhanced (DCE)-MRI in HD patients reflects increased BBB permeability in the caudate nucleus as well as an increase in the gray matter cerebral blood level.

#### Parkinson’s disease (PD)

PD is characterized by the accumulation of oligomeric α-synuclein (α-syn), and dopaminergic neuron degeneration observed in the part of substantia nigra- pars compacta (SNpc) which further prompts motor impairments [116]. Since pars compacta follows a nigrostriatal pathway that contributes in stimulating the cerebral cortex as well as initiating movement, the degeneration of pars compacta neurons ultimately results in a low movement state. The PD patients experience muscular stiffness in the limbs causing difficulty in normal walking, running, etc. Other symptoms are dizziness, cognitive impairments, dementia, loss of postural reflexes, and reduced facial expression.

**Fig. 6:**
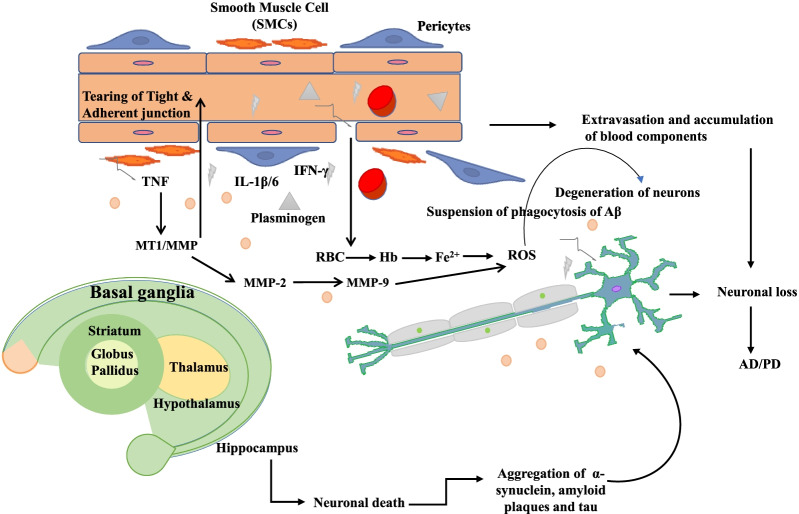
Key pathological characteristics of Parkinson’s disease (PD) are depicted. These include cell death in the Substantia Nigra and Lewy bodies [aggregation of α-synuclein (α-syn),] affecting dopamine releasing neurons. BBB breakdown leads to accumulation of various blood components, leading to production of ROS and thus affecting dopaminergic astrocytes and neurons in case of PD. This figure is adapted from [118]

Vascular dysfunction of the basal ganglia in PD patients leads to breakdown and dysfunction of BBB. In a study, Magnetic resonance imaging (MRI) demonstrated microbleeds, and diminished active efflux of xenobiotics as well as other potential toxins being  reported by verapamil-PET [54]. Besides, increased ^11^C-verapamil uptake in frontal white matter regions was observed in comparison to controls [117]. BBB breakdown leads to the accumulation of neurotoxic fibrinogen, thrombin, plasminogen, and RBC extravasation. The release of Hb and Fe^2+^ generates ROS harmful to dopaminergic neurons. Proinflammatory cytokines like IL-1β, IFN-γ, and TNF as well as various MMPs, etc., released during neurodegeneration are shown in Fig. 6. Few recent studies highlighted the ability of α-syn to cross the BBB and its contribution in the accumulation of α-syn pool in the brain, further clearance from the brain across the BBB occurs through LRP1-mediated transcytosis.

Missense mutations in DNA variants such as LRRK2 (Leucine-rich repeat kinase 2) have been associated with late-onset PD (> 50 years) [119]. Additionally, in vivo studies highlighted MDR1 mutations associated with familial as well as sporadic PD. MDR1 which is highly expressed at the BBB endothelium encodes for ABCB1 (P-glycoprotein) which shows decreased expression in the case of PD patients and leads to progression of PD [117]*.*

#### Multiple sclerosis (MS)

Multiple sclerosis (MS) is a neurodegenerative disease that disrupts the BBB and eventually allows CD4^+^ T cells, peripheral macrophages, and B cells to enter into CNS. This triggers an inflammatory cascade that results in axonal loss and demyelination [120]. MS leads to demyelination of the nerves and causes inflammation, affecting the spinal cord, brain, and optic nerves of the CNS. Several genes of the Major Histocompatibility Complex (MHC) make the individual susceptible to MS. It has been considered that leukocytes, particularly the T-cells migrate to the BBB [121]. The symptoms include disturbances in motor, sensory, bladder and bowel, optic nerve functions, cognitive impairments, tingling, numbness, vision problems etc. An early BBB breakdown, fibrinogen accumulation, reduced expression of TJs, and endothelial degeneration are the characteristic features of MS. BBB dysregulation and trans-endothelial migration of activated leukocytes are among the earliest cerebrovascular abnormalities observed in MS, which induces inflammatory cytokines/chemokines release [122]. Activation of cerebral endothelial cells (CECs) by Th1 cytokines changes occludin and vascular endothelial cadherin (VE-Cadherin) phenotypes in TJs and AJs of CEC via several inflammatory genes [66]. Since most of the drugs are unable to penetrate the BBB, no effective drug against MS could be designed to date. However, a few years ago, the discovery of a peptide molecule, dNP2 which is permeable to the barrier in conjugation with cytotoxic T cells proved to be effective against the disease [123].

## Autoimmune neurological diseases

A significant number of diseases, including auto-immune diseases associated with the CNS have been observed to share similarities; these disorders breach the BBB.

### Susac’s syndrome

It is a rare disorder involving CNS disruption, hearing loss, and branch retinal artery occlusion (BRAO) leading to vision damage. Lesions are commonly observed upon examination by MRI. Several neuropsychiatric symptoms are linked that include personality disorders. Symptoms like headache, confusion, mood changes, seizures, etc. are observed. Further, there may be a complete vision loss, blurred vision, photopsia, etc. [124–126]. This syndrome is usually found to be more prevalent among women. Patients may recover if this disease is identified at an early stage [126, 127].

### Chronic inflammatory demyelinating polyneuropathy (CIDP)

Chronic inflammatory demyelinating polyneuropathy (CIDP) is a disorder that primarily affects the peripheral nervous system (PNS) and involves demyelination of the peripheral nerves. It is caused due to self-reactive antibodies produced against antigens on the peripheral nerves [128, 129]. CSF protein levels get raised in most of these cases and it is more commonly observed among men. Lower limbs are affected first and weakness causes difficulty in getting up [130]. It has been further categorized into different sub-types depending on clinical manifestations [129].

### Bickerstaff brainstem encephalitis (BBE), Fisher syndrome (FS), and Guillain–Barre syndrome (GBS)

Bickerstaff brainstem encephalitis (BBE), Fisher syndrome (FS), and Guillain–Barre Syndrome (GBS) are the autoimmune disorders. All are related in terms of symptoms and affect the CNS as well as PNS. Moreover, these three disease conditions may even have a common origin, because the autoantibodies present are collective [131]. Patients suffering from FS, BBE, and GBS have antibodies against gangliosides FS usually involves ataxia along with hyporeflexia or areflexia and ophthalmoplegia. BBE is characterized by reduced consciousness, ataxia, and ophthalmoplegia. Disturbed consciousness is a feature specific to BBE and differentiates this disease from FS. GBS primarily involves flexia and symmetrical limb weakness [132].

### Cerebral ischemia

Activation of neuroinflammation causes injury to blood vessels, which in turn, affect the BBB structure and function, resulting in cerebral ischemia [133]. Even a stroke is a form of cerebral ischemia whereby the reduced blood supply to the brain can initiate neurological diseases. Inflammation brings immune cells to action, releasing cytokines and other effector cells that reestablish tissue integrity. Excessive microglial cell activation causes the release of interleukins, interferons, and TNF-α due to leakage of BBB, which may further cause neuronal death [134–136].

### Neuromyelitis optica (NMO)

Neuromyelitis optica (NMO) damages the optic nerves as well as the spinal cord by the development of lesions. It is an auto-immune CNS disorder. Specifically, the patients have serum IgG antibodies against aquaporin 4, which is a water channel aquaporin. The antibodies on entering the CNS bind with the aquaporin 4 of the astrocytes, leading to activation of the classical complement pathway [137, 138]. Several factors that promote BBB access have been suggested to participate in the formation of such antibodies.

### Neuropsychiatric systemic lupus erythematosus (NPSLE) and Sjogren’s syndrome

NPSLE and Sjogren’s syndrome lead to the development of autoantibodies and self-reactive lymphocytes. NPSLE (inflammatory auto-immune disease) has symptoms very similar to other neuropsychiatric disorders. Neuropsychiatric symptoms associated include seizures, aseptic meningitis, cognitive disorder, confusion, and psychosis [139]. It often leads to dysfunction of BBB which is further involved in disease progression. Besides, tissue damage and neuroinflammation caused due to antibodies and other effectors of the immune system such as cytokines also play contributory roles [140]. Sjogren’s syndrome is an autoimmune disease that causes inflammation of the lacrimal and salivary glands which further leads to the drying of the mucosa [141].

### CNS vasculitis

CNS vasculitis leads to inflammation and necrosis in the CNS blood vessels. A wide variety of conditions like arthritis, encephalopathy and Sjogren’s syndrome are associated with the disease, besides the increased C-Reactive Protein (CRP) and Erythrocyte Sedimentation Rate (ESR) levels. Treatment is done by administration of corticosteroids [142, 143].

## BBB and therapeutic approaches

CNS disorders involve breaching the BBB integrity of the BBB and its repair is important for the treatment of the aforementioned diseases. In neurological disorders like MS, MMP-9 along with urokinase-type plasminogen activator (uPA) levels are increased, which may damage the BBB. The administration of methylprednisolone that targets the MMP-9 gene and functions by closing the BBB has been noted to be effective [144]. Minocycline, an antibiotic medication is likewise used to repress MMPs for treating stroke. It works by inhibiting microglia activation post-stroke [145]. Since BBB prevents a majority of the drugs and other restorative molecules from gaining access to the CNS, identification of drugs with better penetrability across the BBB is a prerequisite [66]. Steroids are generally used to treat infections in the CNS and work effectively yet the dosage should be carefully managed. Glucocorticoids (GCs), a class of steroid hormones came forth as potent drugs against BBB dysfunction. These serve to protect against edema, inflammation, and other CNS disorders like MS and tumors. These bind to corresponding glucocorticoid receptors (GRs), which are abundantly present in glial cells and neurons [146, 147]. GCs aid in BBB maintenance by enhancing the expression of TJs and AJs that strengthen the barrier [146]. Furthermore, steroids like progesterone and estrogen have protective roles in terms of reducing oxidative stress, edema, and inflammation, besides preventing apoptosis and maintaining homeostasis. A proposed mechanism of action is the regulation of aquaporin-4 which is considered to be important for the development of edema after an injury to the brain. These hormones protect the CNS as has been understood through several studies carried out on neurodegeneration [148–150]. CNS cells like astroglia possess receptors for these steroid hormones. Such properties of these compounds can be exploited for the treatment of multiple neurological disorders [148].

Allopregnanolone, a metabolite of progesterone has been reported to be better as observed in several models [151, 152]. MMP-9 and MMP-2 levels have been seen to lessen on the administration of progesterone and allopregnanolone, while the levels of claudin 5 and occludin 1 were altogether improved [153]. Cyclosporine A is also known to target MMP-9 in experimentally induced subarachnoid hemorrhage. Improved outcomes after the CspA administration were evaluated by several tests [154]. Drugs like glatiramer acetate are effective for treating diseases like MS and for exploratory autoimmune encephalomyelitis (EAE). It is an immunosuppressive drug sold under the name Copaxone which activates on binding with class II MHC molecules and inhibits immune responses. It reduces the B-cell population besides affecting cytokine production [155, 156].

## Endothelial transport mechanisms

**Fig. 7:**
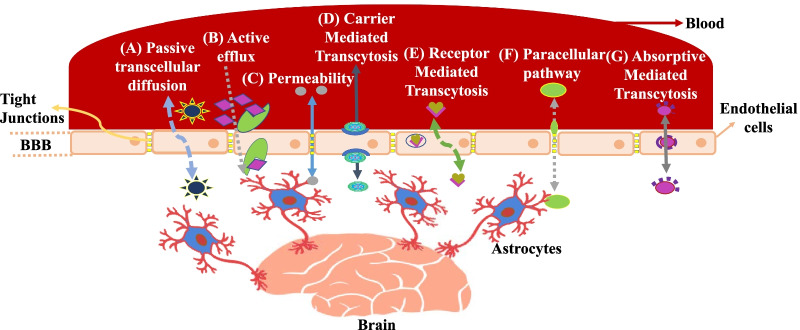
Transport pathways/routes allowing accessibility across BBB. **A** Passive transcellular diffusion/dispersion: Passive diffusion of solutes across BBB is facilitated by higher solubility of lipids. **B** Active efflux of penetrating solutes out of ECs is mediated via efflux carriers **C** Modulation of TJs affects the paracellular diffusional pathway permeability. **D** Carrier-mediated transcytosis system transport several essential polar molecules into CNS like glucose, nucleosides, etc. **E** Macromolecules such as proteins and regulatory molecules, across the cerebral endothelium can be directed via receptor mediated transcytosis. **F** Paracellular pathway is used by small water-soluble molecules for movement across CNS. **G** Adsorptive mediated transcytosis is induced by cationic macromolecules which aid movement across BBB. The idea of the figure is adapted from [164]

Treatment of CNS diseases still remains a challenge for the scientific community. BBB remains the preliminary physiological barrier that limits brain accessibility. Few protein transporters, signaling molecules, and others such as efflux transporters, ion directed channels, etc. are present to ease the transport of several metabolites into the brain [157]. The discovery of a potential vehicle system for CNS drug delivery requires better comprehension of the BBB physiology, its nature, and various transport mechanisms. The entry of drugs, toxins, pathogens, or any other foreign components to the CSF can be prevented by BCSFB. The choroid plexus is the primary constituent of BCSFB, inclusive of choroidal and epithelial cells; it acts as a combination of physical barrier, strategical immunological barrier, and enzymatic barrier to facilitate the transport of drugs, metabolism, and signaling functions [158]. The diagrammatic representation of transport mechanisms is given in Fig. 7. In addition to various barriers that restrict the entry of several substances across the brain, some other provisions are present in the highly selective semipermeable membrane which help the transport of necessary materials in the brain, like the polar or hydrophilic components via the paracellular pathway, whereas small lipophilic substances are carried through transcellular pathway [157].

Other active transport processes that mediate drug permeation and transport of essential nutrients via BBB include adsorptive transcytosis (for instance, transport of albumin), efflux transport, receptor-mediated transport (RMT) and carrier-mediated (through GLUT-1 protein) transport. Transport of various molecules via adsorption can be carried out by caveolar or clathrin-coated membranes. For adsorptive transcytosis, interaction between ligand and moieties on the brain ECs is a prerequisite. It can either be a specific or non-specific process. Caveolae and coated pits both facilitate transcytosis of different molecules. Different studies have provided evidence that adsorptive transcytosis as well as RMT are concentration and time-dependent and need energy for carrying out the processes [159]. The transport is slower in comparison to the ones mediated via carriers, for instance, the transport of glucose [160]. Nevertheless, both RMT as well as adsorptive transcytosis are ideal for macromolecular transport [159]. RMT is among the recently explored physiological process of transcytosis which helps to transport cargo through the ECs toward brain parenchyma. Receptor-mediated transcytosis aids in understanding receptor binding, intracellular trafficking as well as protein engineering, thus enhancing the possibilities for the treatment of CNS diseases [161]. The BBB consists of several systems for carrier-mediated transport of small molecules in order to protect the CNS. For instance, the transport of glucose and amino acids from blood to brain via influx systems. These influx transporters supply essential nutrients to the brain. Therefore, the drugs capable of effectively imitating the substrates of influx transport would have higher probability of crossing the BBB [162]. These transport mechanisms not only help in transporting hydrophobic moieties and various drug molecules from the brain to the blood but also regulate trans-endothelial migration of pathogens and circulating blood cells [163].

EC membrane mediates the bi-directional movement of polar molecules (solute carriers (SLCs). SLCs facilitate the transport of key nutrients such as glucose, amino acids, nucleosides, organic ions, and also a few drugs like L-DOPA, etc. into the brain. Moreover, ATP is not required by SLC transport systems as they are driven by ion-coupled transporters, facilitated transporters, and exchangers. In the case of the facilitated transporters, the difference in the electrochemical potential is utilized for the transport of the SLCs, whilst ion-coupled transporters use proton or sodium gradient directing the transport of SLCs against the concentration gradient ultimately; EC membrane transports the SLC by these transporters. These transporters are present throughout the BBB, blood-testis barrier, liver, intestine, choroid plexus, kidney, and placenta [165].

The carrier-mediated transport mechanisms protect the BBB significantly. Solute molecules comprise of large molecules which are lipid-soluble. These molecules have a strong affinity for ABC transporters which employ ATP hydrolysis to transport molecules to the membrane transversely, thereby against the concentration gradient, the efflux of the solute is ensured [166]. The proteins of the ABC transport superfamily are arranged into seven different sub-families A–G. Sub-families of ABC transporters particularly MDR1 can bestow resistance to cytotoxicity and targeted chemotherapy and also have a significant functional transport role in BBB and BCSFB. For instance, the MDR proteins (viz., P-glycoprotein and BCRP) expressed by ECs further function as ABC efflux transporters.

Receptor-mediated transport system (transcytosis) (RMT) with the help of the ECs transport system, transports substrates on the ECs luminal side. The mechanism of transport via RMT involves binding with a number of macromolecules like transferrin receptor (TfR), insulin receptor protein, etc. Additionally, nutrients like insulin, leptin, and iron are distinctively transported by an endocytic pathway which is referred to as transcytosis [167].

## Novel strategies for effective brain targeting

**Fig. 8:**
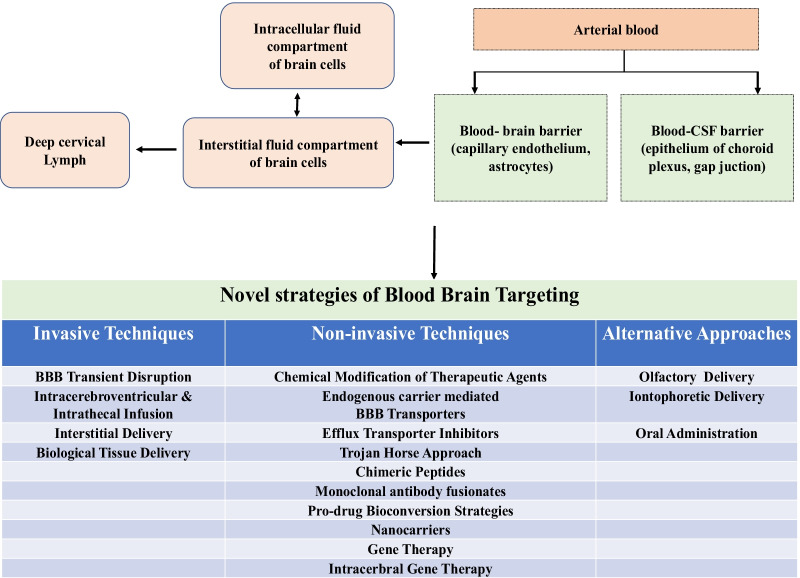
Various novel strategies for effective drug delivery into the brain: invasive techniques, non-invasive techniques and other alternative approaches adapted from [170]

Effective treatment approaches against brain disorders like AD, PD, depression, epilepsy, schizophrenia, and migraine are not available yet. There are several barriers/difficulties in drug delivery trials, one among them being poor drug penetration against neuronal targets. For effectively targeting the brain using bioactive substances, various novel strategies that include invasive systems, non-invasive/miscellaneous systems and various alternative routes for drug delivery into CNS have been designed and are shown in Fig. 8. Various novel strategies and models of transport for the delivery of drugs across the BBB are discussed in Table 1.

**Table 1:** Novel strategies and various popular transport models to deliver therapeutic drugs through the BBB

Novel strategies	Route	Merits	Demerits	Drugs/molecules	References
Osmotic disruption	Paracellular	• Transient • Alters barrier-inducing factors • Promising delivery for recombinant vectors	• Invasive • Transient cerebral edema • Non-specific	• Anticancer drugs • Cytotoxic drugs • Adenoviral vectors	[188, 189]
Chemical disruption	Paracellular	• Transient • Site-specific drug delivery	Conflicting results in clinical trials	• Neuropeptides • Neurotransmitter • Antibiotics • Antineoplastic agents	[168, 190, 191]
Biochemical disruption	Non-invasive	Selective opening of brain tumor capillaries	Breaks down the self-defense mechanism	• Intracarotid infusion of leukotriene C4	[192]
Evade active efflux	transcellular	Involved in multidrug resistance	Restricts the drug distribution	• Phenothiazines • Inhibitors of serotonin re-uptake	[193]
Tight junction pathways	Paracellular (diffusion)	A high capacity pore pathway	Require novel high resolution techniques to detect single openings and closings	Activation of apical sodium-glucose cotransport (SGLT1)	[193]
Nanoparticle delivery	Paracellular (diffusion) and Transcellular (transcytosis)	• Targeted • Sustained and/or regulated release	• Expensive • High toxicity • Clinical efficacy undemonstrated	Liposomal doxorubicin, temozolomide	[194–196]
Biodegradable polymer	Encircle BBB	• Controlled drug delivery	• Useful for limited patients		[164]
Pro-drug		• High drug residence time • Specific membrane transporter	• Low selectivity • Low retention • Toxicity	• Fatty acids, glyceride or phospholipids • Precursor ofGABA, • Niflumic acid • Valproate or vigabatrin	
Biological tissue delivery	Invasive	• Co-grafted cells release therapeutic proteins	• Inefficient transfection • Non-selective expression • Deleterious regulation	Intracarotid infusion of leukotrienes, bradykinin	[169]
Vasoactive peptides	Transient	Non-invasive	• Poor clinical efficacy	RMP-7/ labradimil/Cereport	[197, 198]
Cell-mediated endocytosis	Transcellular	Targeted	• Toxic for cell carrier system • Less therapeutic loading	• TAT • Penetratin • polyarginines	[199–201]
Focused ultrasound	Paracellular and Transcellular (diffusion and convection)	• Non-invasive • Targeted	Costly	• Antibodies, doxorubicin • Carboplatin	[202, 203]
Radiation	Paracellular and Transcellular	• Increases permeability	Radiation-induced (Neuro) inflammation	Insulin	[204, 205]
Intrathecal and intraventricular delivery	Bypass BBB	• Encounter minimized protein binding • Decrease enzymatic activity • Longer drug shelf-life	• Invasive • Low parenchymal concentrations • Prompt CSF turnover • High clinical incidence of hemorrhage • Neurotoxicity	Recombinant human heparin-N-sulfatase (rhHNS)	[206–208]
Olfactory pathway	Crosses BBB	• Non-invasive • Simple drug administration	• Discomfort nasal mucosa • Lower efficiency	Neurotropic factor	[209]
Interstitial wafers, microchips and nanospheres	Crosses BBB	• Sustained and controlled release • Easily implantable without damage	• Invasive • Distribution is limited through ECS		[210, 211]
Convection- Enhanced Delivery (Injections, Catheters and Pumps)	Bypass BBB Through transcellular	• Enhances distribution by bulk flow	• Invasive • backflow of infusate • catheter misplacement risk • Expensive • Low efficiency	• Gadolinium • Magnetic nanoparticles	[198, 212]
Carrier-mediated	Transcellular (transcytosis) Non-invasive	Controls the delivery and retention of drugs	A highly stereospecific drug is converted to structure similar to that of endogenous nutrient	Levodopa • Melphaling • Glucose	[213]
Receptor-mediated	Transcellular (transcytosis)	• Allows planned transport linkers to suit the characterized functional requirement of the therapeutic agent, including peptide-based pharmaceuticals and small molecules incorporated within liposomes • These transporters can be examined for brain delivery	Saturable process, enzymatic drug release, attachment to a BBB transport vector depicts certain drugs inactive	• Transferrin receptor • Lactoferrin receptor • Insulin receptor	[214–216]
Adsorptive mediated	Transcytosis	• Uses a cationic biological macromolecule		• Cationized bovine serum albumin (BSA) • Cationized immunoglobulins/monoclonal antibodies (Mabs)	[215, 217]

### Invasive techniques

#### BBB transient disruption

Blood–brain barrier disruption therapy (BBBD) is emerging as an effective approach for the delivery of therapeutics for brain tumors. Using this method, the desired amount of therapeutic drug can be delivered to the tumor and other adjacent tissues. Further, BBB disrupts transiently by breaking down the TJs so that it can permit transport of various molecules, ultrasounds (trans-skull focused ultrasound (FUS), MRI, or hyperosmotic solutions (like mannitol, arabinose, lactamide, saline, urea, DMSO, ethanol, glycerol, polysorbate-80, and X ray-irradiation, etc.). However, this technique has several limitations; for instance, it  disupts the integrity and physiological functions of the BBB, which leads to possible undesirable aggregation of blood components, xenobiotics, and exogenous agents in the BBB by inducing CNS injury [163].

#### Intrathecal and intracerebroventricular infusion

For quite some time, intracerebroventricular (ICV) devices have been utilized in the treatment of a wide assortment of pediatric and adult CNS disorders. CNS diseases require the direct administration of medications into the brain to accomplish full remedial impact. In any case, this physiological hindrance limits the movement of colossal molecules in between the blood, CSF, and interstitial fluid of the brain. To resolve this issue, intrathecal delivery strategies are used that regulate soluble therapeutics directly into the CSF. Intrathecal delivery strategies incorporate ICV, intrathecal-lumbar and intracisternal courses. The ICV route empowers the administration of medications into a lateral cerebral ventricle. Further, repeated administrations of therapeutic drugs are required to improve its proficiency and improve the rate of clinical success.

#### Interstitial delivery

Drug delivery through intrathecal and intracerebroventricular strategies elude the BBB to some point, the direct administration of therapeutic drugs into the interstitium is the most direct strategy of brain targeting delivery systems [168]. BBB has restricted the adjuvant treatment of brain tumors with chemotherapeutic agents introduced systemically. This physiological and pharmacological boundary is due to the presence of TJs in between the  ECs of the capillaries of the CNS. As a rule, only minute, lipid-soluble particles that are electrically neutral can infiltrate this capillary endothelium. Most of the chemotherapeutic agents are not included in this category. However, a couple of cytotoxic agents, for example, the nitrosoureas, are observed to be effective in treating brain tumors. These agents have been transported systemically in high doses to attain restorative levels in the CNS. This methodology unassumingly affects the endurance of patients with malignant gliomas, however, has additionally resulted in systemic aftereffects.

To overcome these issues, the chance of delivering chemotherapeutic agents interstitially inside the brain parenchyma utilizing controlled delivery polymers has been investigated. This methodology has two significant benefits. The primary benefit is that interstitial drug delivery avoids the BBB. Utilizing this methodology, any medication  incorporated into polymer is possible to administer into the CNS. The subsequent benefit is that this type of drug delivery can bring about significant degrees of drug concentration at the site of pathology with negligible spillage of the medication into the systemic circulation. Therefore, the undesirable effects of a cytotoxic agent could be reduced.

#### Delivery to tissues

An alternative strategy for interstitial delivery of the drug necessitates drawing out the therapeutic drugs from biological tissues. This technique involves tissue implantation into the brain, which secrete desired therapeutic agent naturally. This method is widely used for treating PD patients [169]. However, the survival of foreign tissue grafts is the only major drawback of this technique wherein the transplanted tissue is not capable of surviving due to the absence of neovascular stimulation.

### Non-invasive techniques

This strategy involves drug modification pharmacologically to alleviate the drug delivery across the BBB. Different non-invasive techniques are discussed as follows:

#### Drug modification for enhancing its lipid solubility

As passive diffusion is the fundamental mechanism for the delivery of lipid molecules across the BBB, lipid solubility is a key factor. To overcome this barrier, the chemical modification of therapeutic agents into a lipophilic form is done through process of lipidization and can be modified by adding lipid or functional groups into lipid-soluble agents which can pass through the BBB [169]. The benefits of lipophilic analog delivery into the brain might countervail the change of drug pharmacokinetic parameters [171].

#### Use of carrier-mediated transport systems

The chemically modified therapeutics (small-molecular drugs) can be used as endogenous transport/carrier systems. The widely used carrier-mediated BBB transporters are small molecules that mimic the structure of specific endogenous molecules present in the brain for effective drug delivery. Examples of different small molecules are monocarboxylic acid [monocarboxylic acid transporter type 1(MCT1)], monosaccharides [glucose transporter type 1 (GLUT1)], acidic amino acids [cationic amino-acid transporter type 1(CAT1)], neutral amino acids (large neutral amino-acid transporter type 1(LAT 1)), vitamins, hormones, purine bases, and nucleosides [equilibrative nucleoside transporter 1 (ENT 1)], etc. [163, 172].

#### Efflux transporters for drug delivery

Efflux transporters can function as vacuum cleaners of xenobiotics in the endothelium of the cerebrovascular system, which impede the drugs destined to their target places. The improved cell accumulation of therapeutic drugs can be achieved by efflux transporter inhibitors. The prohibition of efflux transporters selectively hinders the transport of therapeutic drugs to BBB. A few examples of efflux transporters include phosphorylated glycoproteins, BCRP, and MDR in both humans and rodents [165, 173, 174].

#### Receptor-mediated Trojan horse approach

Trojan horse approach is being widely used for the delivery of therapeutic carriers/vectors, non-viral remedial drugs, and proteins through BBB into the brain. Generally, the monoclonal antibodies or endogenous molecules act as trojan horses by binding to the epitopes of the receptor-mediated carrier systems of the BBB by intruding onto the receptors and thereafter to the therapeutic drugs. Finally, after binding trojan horse on to the therapeutic medicine, it diffuses into the brain parenchyma whereas the receptor moves back to the membrane to carry another therapeutic molecule to the BBB [175].

#### Delivery of chimeric peptides

This technique involves using peptides or proteins of pharmaceutically non-transferable to transferable peptides through the BBB with the help of transcytosis pathways (receptor-mediated or absorptive-mediated transcytosis). One of the most inventive methods for trans-endothelial cellular transport involves the regulation of the endogenous RMT pathway [168].

#### Re-engineering of monoclonal antibody fusion proteins

The re-engineering of recombinant monoclonal antibodies or trojan horses for the targeted brain drug delivery has been shown to improve efficiency in the ongoing mouse models of neurological disorders like PD, AD, and lysosomal storage disorders [176].

#### Nanocarrier-based technologies

The nano-sized (1–100 nm) molecules improve the transportation of drugs across the BBB because of their smaller size and surface functionalization with the target-specific moieties [177]. Diverse nanocarriers like liposomes, polymers, dendrimers, quantum dots, inorganic nanoparticles, nano gel, and nano-emulsion, etc. are available based on the type of nanoparticle used, method of preparation, drug loading, and release behavior of the drug [178].

#### Gene therapy

This technique involves the direct transfer of therapeutic recombinant DNA into cells of the targeted site and involves both ex vivo and in vivo methods by either therapeutic gene transfer directly into the patient or using gene carriers such as vectors (viral or non-viral). Gene therapy has been suggested to be a potential solution against neuropathological disorders [179].

#### Intracerebral gene therapy

The limitations of gene therapy viz., drug dosage efficiency, and toxicity of vectors open up the scope for intracerebral gene therapy. This technique involves the direct transfer of viral gene injection inside the brain parenchymal cells or ventricular system. Several viral systems like adenovirus, retrovirus, adeno-associated virus, and herpes simplex virus, etc., have been explored for drug delivery approach. Due to higher transfection efficiency, many drug delivery systems using virus-like particles and virosomes have been developed [180–182]. Experiments have provided evidences that the adeno-associated virus serotype 9 (AAV9) is capable of delivering cells in the CNS when administered intravascularly to cats, rodents, etc., which implies its ability to cross the BBB. It is also shown to penetrate the ECs in vitro [183]. In vivo analysis has shown functional improvement in various disorders. Delivery of drugs directly into the CNS using the adeno-associated virus-based animal models has further proven the futuristic benefits of this approach [184].

### Different routes for drug delivery to the CNS

#### Intranasal/olfactory drug delivery system

Substitutive drug delivery through the nose to the brain has been found to be an effective approach in the past few years. Researchers claim that the drug can be directly delivered into the brain without entry into the systemic blood circulation [185]. Practically, any drug or dosage enters drop by drop into the nasal cavity which gets absorbed in the respiratory tract. Drug further moves into the systemic circulation, and crosses the BBB via the intraneural pathway which promotes the cellular transport mechanism [186]. The nasal cavity comprises of some trigeminal neurons; therefore, few drugs are carried directly into the brain via trigeminal nerves. Continuous research efforts are being made for improving the efficiency of intranasal drug delivery. For example, significant progress in the pharmacokinetic behaviour and brain targeting efficiency of the lamotrigine-loaded PLGA-nanoparticle for direct intranasal delivery has been reported [187].

#### Iontophoretic delivery

This method involves the delivery of ionized molecules using applied electric current externally across the BBB. Some non-invasive iontophoretic also utilize the intranasal pathway designed for the delivery of drugs into the CNS [169]. In addition to the invasive and non-invasive methods, iontophoresis devices also show increased drug delivery of the macromolecules into the brain under controlled manipulation.

## Nanomaterials for treatment of neurodisorders

Nanoparticles (NPs) are colloidal particles with sizes in the range of 1 to 100 nm [218]. These NPs are comprised of micromolecular constituents in which the drug or some other biologically active molecules are encapsulated, entrapped, adsorbed, or attached. The sources of origin for nanoparticles can be natural or synthetic. The designing of NPs which are compatible for drug delivery across BBB involves certain parameters. These parameters include stability of biological fluids, biodegradability, favorable pharmacokinetic properties, adequate release profiles, non-toxicity, biocompatibility, and good drug loading efficiency [219, 220]. Different types of NPs that are designed for drug delivery include polymeric NPs, lipid-based NPs, and inorganic NPs.

### Lipid-based NPs

**Fig. 9:**
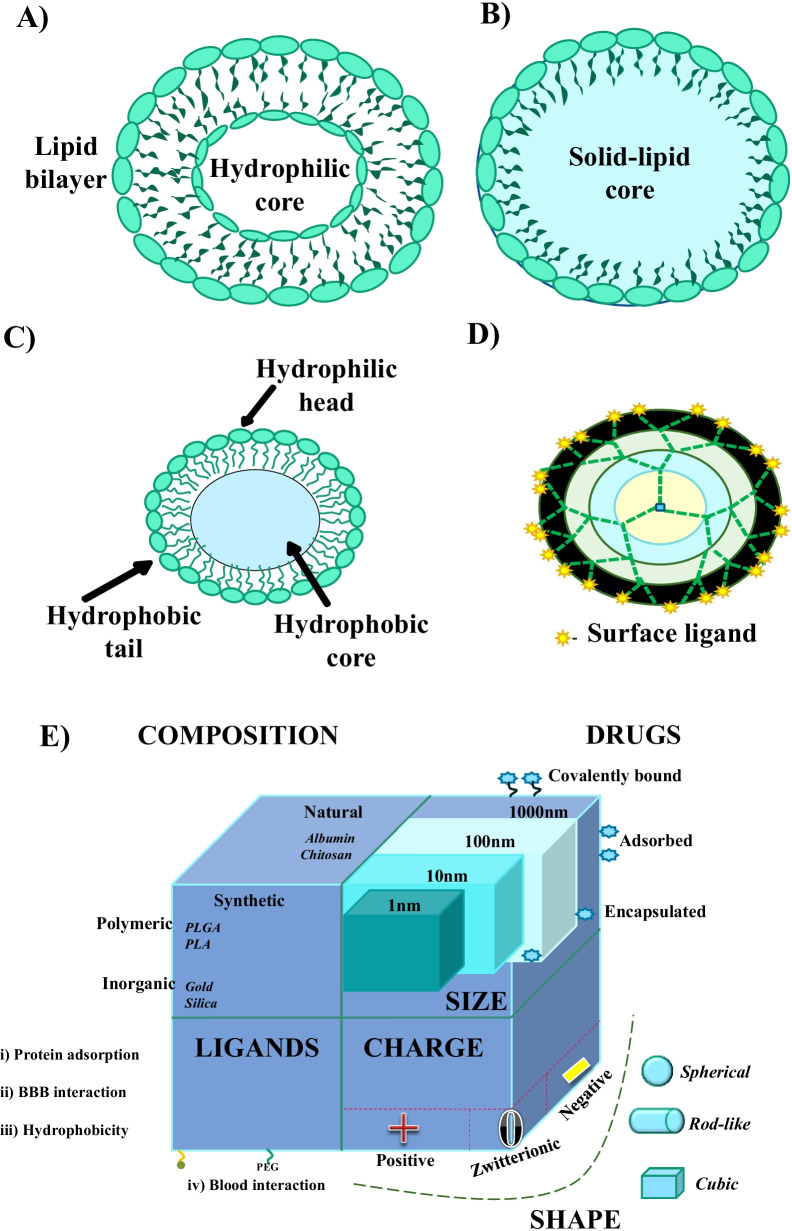
Lipid-based nanoparticles for treating neurodisorders are illustrated **A** liposomes, **B** solid lipid nanoparticle (SLN); Polymeric nanoparticles, **C** polymeric miscelles, **D** dendrimers and **E** illustrating the major properties of nanoparticles that influence systemic delivery and transport through BBB. NPs have the ability to deliver drugs into cells by covalently bounding, entrapping or adsorbing them. They can be of different shapes (rod-like, spherical, or cube) and charges (positive, zwitterionic, or negative). NPs can be natural such as proteins (albumin), chitosan or synthetic NPs which are made from commonly used polymers like poly (lactic acid) (PLA), poly (lactic-co-glycolic acid) (PLGA), or from inorganic agents like gold, silica, or alumina. Also, these NPs can be functionalized using different types of ligands. (i) efficient in mediating protein adsorption [Polysorbate-80 (P-80)], (ii) direct interaction with BBB (transferrin proteins, peptides or antibodies), (iii) increasing hydrophobicity (amphiphilic peptides), and (iv) ability to increase blood circulation (PEG). The figure is adapted from [235]

Lipid-based NPs are known to be stable and possess less toxicity for in vivo applications. The most common types of lipid-based NPs include liposomes and solid lipid nanoparticles (SLN). Liposomes are spherical vesicles comprised of a lipid bilayer with an internal aqueous space [221, 222]. These are predominantly comprised of amphiphilic phospholipids like sphingomyelin, phosphatidylcholine, and phosphoglycerides (as shown in Fig. 9A). Cholesterol is additionally incorporated to increase the stability of liposomes and forestall the leakage of bilayer [4]. Liposomes are subcategorized based on the number of lamellae and on the basis of their size a. small unilamellar vesicles (SUVs) having sizes up to 100 nm and single lipid bilayer b. large unilamellar vesicles (LUVs) of sizes greater than 100 nm, and the single bilayer and c. multilamellar vesicles (MLVs) greater than 500 nm and have several concentric bilayers. Based on formulation, liposomes can be categorized as anionic, cationic, or neutral [223, 224]. There are certain drawbacks related to liposomes which include complex preparation procedures, low physical stability, and controlled as well as sustained drug release [225].

SLNs (as shown in Fig. 9B) are stable, spherical lipid-based nanocarriers, having a solid hydrophobic lipid matrix which can successfully entrap various lipophilic molecules. The core matrix is made up of waxes, fatty acids, triglycerides, and steroids, and is stabilized through surfactants. SLNs range from 40 to 200 nm in size which allows them to cross the tight ECs of the BBB [226]. In vivo studies suggested that the SLNs modified by surface functionalization, for specific targeting of the brain and reticuloendothelial system (RES), significantly increased the distribution of neuroprotective resveratrol in the brain. The SLNs loaded with resveratrol functionalized with APOE and these can be further recognized by low-density lipoproteins (LDL) receptors present on the BBB [227, 228]. The advantages of SLN include their biocompatibility, higher amount of drug entrapment as compared to other nanoparticles, and their capability for continuously releasing the drug for a few weeks [229].

### Polymeric nanoparticles

Polymeric NPs consist of a polymer matrix core in which the desired drug can be embedded for delivery. These NPs have the potential to efficiently deliver drugs to the CNS. Some polymeric NPs used for this purpose include polymeric micelles, and dendrimers, etc. [230].

Polymeric micelles (Fig. 9C) act as nanocarriers and are comprised of amphiphilic copolymers arranged spontaneously in the aqueous solution. These possess a hydrophilic shell as well as a hydrophobic core which allows the loading of hydrophobic drugs in the core [231]. The modification of polymeric micelles provides high stability, higher loading efficiency, and a controlled drug release profile. Additionally, these modifications also improve the bioavailability and solubility of various insoluble drugs [232].

Dendrimers (three-dimensional) branched polymers having spheroidal and symmetrical morphology and size in the range of 1–100 nm (Fig. 9D). The molecular structure of dendrimers is tightly packed at the periphery and loosely packed in the core, leaving spaces that enable entrapment of drug. Dendritic molecules are generally split into high-molecular weight or low-molecular weight species [233]. The former category consists of dendronized polymers, hyperbranched polymers, and polymer brush while the latter comprises of dendrimers and dendrons. Additionally, water-soluble dendrimers can be prepared, in contrast to most polymers, via functionalizing the external shell with the help of hydrophilic groups or charged species. The toxicity of these drug delivery systems can be effectively controlled for in vivo experiments [234].

### Inorganic nanoparticles

Inorganic nanoparticles include superparamagnetic iron oxide nanoparticles (SPIONs), upconversion nanoparticles (UCNPs), quantum dots (QDs), gold nanoparticles (AuNPs), etc. They possess unique electric, magnetic and optical properties for several biomedical applications like targeted drug delivery, biosensing, bioimaging, and cancer therapy (Fig. 9E). Inorganic NPs which include gold, silica, alumina, carbon, and cadmium-based fluorescent particles can be tuned for their shape, structure, composition, size, and porosity. These NPs also facilitate the ligand-polymer conjugation which further enhances their functioning [236]. The conjugation of compounds that could facilitate active transport across BBB is necessary, as they cannot passively diffuse through the BBB for applications in neuro nanomedicine [237]. QDs are nanocrystals of inorganic semiconductors, which have a diameter between 1 and 20 nm possessing luminescent properties. These QDs are generally made of atoms from different groups of the periodic table like groups II and VI elements (CdSe and CdTe) or groups III and V elements (InP and InAs) [238]. Additionally, carbon nanotubes are widely used carbon-based NPs that are composed of graphite sheets rolled into tubes having diameter in the nanometers range [237]. They have a high surface area with unique electrical, optical, mechanical, and thermal properties. The main applications of these nanotubes are in the field of tissue engineering, biosensing, gene therapy, drug, hormone, and enzyme delivery [239]. Similar to carbon nanotubes, QDs require surface functionalization for specific brain targeting and crossing BBB. They have applications in drug delivery, medicine, bio-imaging, cancer therapy, and labeling and tracking of transplant cells [240].

## Nanoparticles for treating neurological diseases

NPs have been widely used for the purpose of diagnosis and effective treatment of various neurodegenerative diseases. The BBB which protects the brain and maintains homeostasis hinders the delivery of NPs into the CNS. Therefore, NPs are specially designed to facilitate their transport across the BBB and provide therapeutic effects against certain neurological diseases like AD, PD, stroke, and brain tumors [241].

Neuroprotective peptides can be used for the treatment of AD; multifunctional NPs act as nanocarriers to deliver those peptides and protect them from being degraded in plasma by proteolytic enzymes [242]. For example, poly(ethylene glycol)-poly(dl-lactic acid) PEG-PLA NPs (a polymeric NP) have been used to carry a neuroprotective peptide NAPVSIPQ and protect it from degradation [243]. Many researchers have performed in vivo experiments using different types of drugs in association with nanoparticles. For example, nicotine-encapsulated poly (lactic-co-glycolic acid) (PLGA) NPs have been effectively used for treating PD patients and were injected intraperitoneally [244]*.* In a study, the development of lactoferrin-conjugated PEG-PLG NPs administered intravenously has been reported [245]. Similarly, another delivery system from the nose to the brain has been proposed using odorranalectin-conjugated PEG-PLG NPs [246]. Therefore, depending upon the physical properties and concentration of drug-associated NPs, they show different acceleratory or inhibitory effects on the fibrillation process.

Further, for the treatment of MS, doxorubicin conjugated liposomes have been used as nanomedicines. Studies have provided evidence that these nanomedicines can lead to better recovery of the disease. SLNs have been used for the delivery of FDA-approved dimethyl fumarate for the management of relapsing MS [247].

## Conclusion and future perspectives

Remedial delivery of various therapeutic agents to the brain is frequently impeded due to BBB, which is the chief obstacle in the treatment of CNS disorders. Future endeavors need to focus on overcoming challenges associated with the BBB and on discovering novel strategies which can effectively deliver drugs into the brain. Recent research has shown that liposomal nanocarriers and biodegradable polymers can successfully be used as a potentially beneficial strategy for neurotherapeutics.

Several molecules/pathways play crucial role in proper functioning of the BBB. The Wnt/β-catenin signaling pathway is a significant regulator for the development of BBB and its maintenance [248]. Unc5 regulates axon guidance in many species and a member of its family, Unc5B is found in ECs of humans [249]. Unc5b interacts with β-catenin in ECs for maintaining integrity of BBB. Unc5B is known to bind Netrin-1 and other ligands via its extracellular domain and its deletion in mice leads to leakage of BBB and reduced Wnt/β-catenin signalling. It has been recently proved that the blocking the binding of Netrin-1 with Unc5B and delivery of monoclonal antibodies prompts the opening of BBB for various molecules transiently. This would pave way for delivery of therapeutics for various neurological disorders [250]. Wnt 7a and 7b are the signals that induce the formation of the BBB and are also involved in BBB repair. The complex of Wnt 7a/b with G protein–coupled receptor (Gpr124) and the glycoprotein Reck can be well utilized for BBB repair with high specificity [251].

Future developments will likely focus on early intervention which can slow down the progression of neurological ailments, since BBB dysfunction and breakdown results in neurodegeneration. Some insights into the molecular mechanism causing these disorders have been gained from various experimental animal models, but the precise mechanism responsible for BBB breakdown still remains elusive. Future research should focus on unraveling details on the dynamics of the BBB, and how alterations in BBB affect the nervous system, further leading to various disorders. A better comprehension of different aspects of BBB dysfunction would aid the development of potential therapeutics against neurodegenerative diseases. Efforts ought to be made for development of strategies to reverse BBB damage. Developing advanced brain imaging techniques capable of detecting changes in BBB integrity would be a promising approach in the field of human neuro-vascular research.

## Data Availability

Data sharing is not applicable to this article as no datasets were generated or analyzed during the current study.

## Abbreviations

NPs: Nanoparticles
BBB: Blood–brain barrier
CNS: Central nervous system
CSF: Blood-cerebrospinal fluid
PD: Parkinson’s disease
AD: Alzheimer’s disease
BCSFB: Blood–cerebrospinal fluid barrier
MS: Multiple sclerosis
BCRP: Breast cancer resistance protein
MRPs: Multidrug resistance-associated proteins
ECs: Endothelial cells
TJs: Tight junctions
AJs: Adherent junctions
CAMs: Cell adhesion molecules
